# Studies on Isolated Tumour Mitochondria: Oxidative Phosphorylation and Fatty Acid Oxidation by Rat Hepatoma Mitochondria

**DOI:** 10.1038/bjc.1961.48

**Published:** 1961-06

**Authors:** P. Emmelot, C. J. Bos


### STUDIES ON ISOLATED TUMOUR MITOCHONDRIA: OXIDATIVE PHOSPHORYLATION AND FATTY ACID OXIDATION BY RAT HEPATOMA MITOCHONDRIA

P. EMMELOT AND C. J. BOS

*From the Department of Biochemistry, Antoni van Leeuwenhoek-Huis: The Netherlands Cancer Institute, Amsterdam, The Netherlands*

Received for publication March 22, 1961

IN a previous paper from this laboratory (Emmelot, Bos, Brombacher and Hampe, 1959) an extensive survey was presented of the metabolic properties of hepatoma mitochondria with special reference to the transplanted rat hepatoma BY 225. This hepatoma was of the solid type and, by lack of evidence to the opposite, was considered to be suitable for a metabolic comparison with the normal rat liver. Meanwhile three other transplanted rat hepatomas have been established, bearing the case numbers BY 446, BY 448 and BY 463. The question of the cell origin of the four rat hepatomas has been discussed in the preceding paper (Emmelot, Hampe, Bos and Reyers, 1961) on account of the levels of deoxycytidylate deaminase activity, an enzyme which was considered by Pitot and Potter (1960) to be a likely parameter for distinguishing between parenchymal and bile duct (tumour) cells. In the present investigation we have been concerned with some metabolic properties, relating to adenosine triphosphate (ATP) production of the mitochondria from the three newly-established hepatomas. These experiments were carried out with a dual purpose in mind. It has been shown repeatedly (Emmelot *et al.*, 1959) that mitochondria from different tumours may behave non-uniformly and, accordingly, it was of interest to investigate, first, to what extent the metabolic properties of mitochondria from transplanted rat hepatomas might vary and, secondly, how far the latter data might furnish information that would allow a classification of the rat hepatomas. Non-uniformity in enzymic behaviour between the hepatoma mitochondria was indeed encountered in the present experiments, one of the hepatomas, BY 448, yielding mitochondria which, in contrast to the other hepatoma mitochondria, were deficient in oxidative phosphorylation and fatty acid oxidation. In view of the fact that of the four rat hepatomas studied, the latter showed consistently the lowest content of deoxycytidylate deaminase activity (Emmelot *et al.*, 1961), we have made some effort to find the reason underlying the difference in mitochondrial activities observed between the hepatomas. Pertinent to this question was the earlier finding (Emmelot *et al.*, 1959) that less satisfactory results were obtained with mitochondria from BY 225 hepatoma transplants which had grown to full size and contained necrosis (the latter was removed; " old " transplants) than with mitochondria from smaller " young " transplants. Accordingly, in the present experiments attention was also directed to the possible effect of transplant size and " quality " on mitochondrial activity. For, if the transplants of the BY 448 hepatomas were to contain more necrosis than those of the other hepatomas or if only " old " transplants of the BY 448 hepatoma had been used, the difference in the mitochondrial activity would have to be ascribed to this artefact. This not being the case, we tried to find some other explanation for the abnormal behaviour of the BY 448 hepatoma mitochondria. In this we were led by the results of other experiments which had shown that the complete removal of the fat droplets from a tumour homogenate was essential in order to obtain mitochondria with a relatively low " free " ATPase activity and a demonstrable DNP-activated ATPase (Emmelot *et al.*, 1959). A second lead was provided by the finding that the *in situ* fine structure of the major part of the mitochondria in liver cells, after the *in vivo* administration of an acutely toxic dose of the hepatocarcinogen dimethylnitrosamine, was not changed (Emmelot and Benedetti, 1960), whereas upon isolation of the particles from such livers an increased lability of their structure and function was noted (Emmelot, Bos, Brombacher and Reyers, 1960 ; Emmelot, Bos and Reyers, 1960). Thus it was considered likely that the mitochondria suffered damage during the homogenisation of the tissue and the subsequent isolation procedure. Since non-structurally-bound fat did accumulate in these livers to a marked extent (Emmelot and Benedetti, 1960) and since fats may damage mitochondria (Pressman and Lardy, 1956 ; Avi-Dor, 1960), the top fatty layer from the homogenates of the treated livers was separated and, after washing, added to normal rat liver mitochondria in order to see whether the properties of the latter might be changed. This fat produced an activation of the latent, $Mg^{2+}$-dependent, ATPase and an inhibition of the DNP-activated ATPase of fresh normal liver mitochondria (Emmelot, Bos, and Reyers, 1960). Similar experiments were, therefore, carried out in the present investigation with the fat obtained from rat liver and the various hepatoma homogenates.

### MATERIALS AND METHODS

The tumours studied in the present investigation have been described in the preceding paper (Emmelot *et al.*, 1961). Transplants, labelled as " young ", were harvested after growth during periods shorter than or equal to those mentioned in the latter paper. Such transplants contained only occasionally necrosis and were then designated as " old transplants ". Otherwise, transplants labelled " old", had been grown for longer periods than those indicated. Necrotic areas, when present, were always removed before homogenisation of the tissues. In view of the fact that the transplants were maintained intraperitoneally, the utmost care was taken to remove the adhering fat as completely as possible. The isolation of the mitochondria (0·25 M sucrose containing 0·001 M ethylenediamine tetra-acetate (EDTA) and the measurements of oxidative phosphorylation, fatty acid oxidation and ATPase activity were carried out as described previously (Emmelot *et al.*, 1959). Non-structurally-bound lipids were separated from the hepatoma homogenates as indicated in the text.

### RESULTS

### *Oxidative phosphorylation*

Table I contains typical data illustrating the oxidative and phosphorylative activities of mitochondria isolated from " young " transplants of the three rat hepatomas BY 446, BY 463 and BY 448 in the presence of glutamate, diphosphopyridine nucleotide (DPN), cytochrome *c*, and the " high energy " phosphate trapping system glucose-hexokinase. Incubation was carried out at pH 7·0 and 7·4 in histidine buffer at 27° C. (Emmelot, Bos, Brombacher and Hampe, 1959). Oxidative phosphorylation by the hepatoma mitochondria, like that by liver mitochondria, was somewhat more efficient at the former than at the latter pH. Earlier experiments had shown that the $Mg^{2+}$-activated ATPase of another transplanted rat hepatoma (BY 227) was less active at pH 7·0 than at 7·4. The P : O ratios obtained with the mitochondria from the hepatomas BY 446 and 463 were satisfactory and, at pH 7·0, equalled that obtained with normal liver mitochondria. However, the efficiency of the oxidative phosphorylation of the BY 448 hepatoma mitochondria was very low. In this respect the latter mitochondria behaved quite differently from the BY 446, BY 463, and the BY 225 mitochondria studied earlier (Emmelot *et al.*, 1959). The specific activity of the mitochondrial

TABLE I.—*Oxidation and Phosphorylation by Mitochondria from " Young " Transplants of the Rat Hepatomas BY* 446, *BY* 448 *and BY* 463, *in the Presence of Glutamate*

Mitochondria were isolated in 0·25 M sucrose containing 0·001 M EDTA (pH 7·4) and incubated (DPN and glucose-hexokinase being added) as previously described (Emmelot *et al.*, 1959).

| Tumour | Transplant generation | Number of transplants used | pH of incubation medium | Mitochondrial nitrogen per flask (mg.) | Time of incubation (minutes) | Oxygen consumption (μatoms) | Phosphate uptake (μmoles) | P : O | Oxygen consumed/mg. mitochondrial N/hour (μatoms) |
|---|---|---|---|---|---|---|---|---|---|
| BY 446 | 13 | 1 | 7·4 | 0·80 | 15 | 7·6 | 12·0 | 1·6 | 38·0 |
| | 15 | 2 | 7·0 | 0·76 | 15 | 6·3 | 14·9 | 2·4 | 33·6 |
| BY 463 | 2 | 1 | 7·4 | 1·25 | 10 | 7·0 | 13·3 | 1·9 | 33·6 |
| | 3 | 2 | 7·0 | 1·01 | 10 | 6·1 | 15·3 | 2·5 | 36·6 |
| BY 448 | 13 | 1 | 7·4 | 0·74 | 15 | 5·1 | 2·3 | 0·4 | 27·2 |
| | 14 | 2 | 7·0 | 0·73 | 15 | 5·0 | 3·5 | 0·7 | 26·7 |

TABLE II.—*Oxidation and Phosphorylation by Mitochondria from " Old " Transplants of the Rat Hepatomas* BY 446, BY 448 *and* BY 463, *in the Presence of Glutamate.*

| Tumour | Transplant generation | Number of Transplants used | pH of incubation medium | Mitochondrial nitrogen (mg. flask) | Time of incubation (minutes) | Oxygen consumption (μatoms) | Phosphate uptake (μmoles) | P : O | Oxygen consumed/mg. mitochondrial N/hour (μatoms) |
|---|---|---|---|---|---|---|---|---|---|
| BY 446 | 12 | 1 | 7·0 | 1·02 | 20 | 3·8 | 0·3 | 0·2* | 11·2 |
| | | | 7·4 | 1·02 | 20 | 5·0 | 0·0 | 0* | 15·0 |
| BY 463 | 5 | 1 | 7·0 | 1·39 | 15 | 5·4 | 8·2 | 1·5 | 15·5 |
| BY 448 | 12 | 1 | 7·0 | 1·05 | 15 | 7·9 | 6·4 | 0·8 | 31·0 |

* In the absence of glucose-hexokinase glutamate oxidation*, expressed as microatoms oxygen consumed/mg. mitochondrial nitrogen/hour, was of the same order of magnitude for all the hepatomas, that of the BY 448 hepatoma tending to be somewhat smaller.

Similar experiments were carried out with mitochondria prepared from " old " hepatoma transplants which contained some necrosis. It is evident from Table II that such mitochondrial preparations from the hepatomas BY 446 and BY 463 gave less satisfactory results than those from the " young " transplants of the same tumours illustrated in Table I. Glutamate oxidation was about half as effective and the P : O ratios were decreased. In the case of hepatoma BY 448 the present results with the " old " transplants were of the same unsatisfactory kind as those obtained with " young " transplants of this tumour ; however, no difference in glutamate oxidation between the mitochondria from " old " and " young " BY 448 transplants could be observed.

The conclusion to be drawn from the results presented in Tables I and II is that " young " transplants should be used for the study of the mitochondrial activities of rat hepatomas and that, if this condition is satisfied, some hepatomas yield mitochondria with an oxidative phosphorylation approximating that of normal liver mitochondria, while other hepatomas may yield particles which are deficient regardless of the fact whether " young " or " old " transplants are used.

TABLE III.—*ATPase Activity of Mitochondria from " Young " and " Old " Transplants of Rat Hepatoma BY* 463

Assay in the presence of $Mg^{2+}$

| Condition transplant (generation) | Mitochondrial nitrogen/flask (mg.) | DNP ($10^{-4}$ M) | μg. phosphorus released after 7·5 min. | 15 min. |
|---|---|---|---|---|
| " Young " (2) | 0·05 | — | 9 | 17 |
| " Young " (3) | 0·16 | — | 20 | 32 |
| | | + | 38 | 66 |
| " Old " (5)* | 0·10 | — | 68 | 110 |
| | | + | 75 | 135 |

* Compare Table V : BY 463¶.

The effect of the age of the transplant (" young " *versus* " old ") is further illustrated in Table III for the $Mg^{2+}$- and DNP-activated ATPase of the mitochondria from the hepatoma BY 463. Although the ATPase activity of these particles (like that of the BY 225 mitochondria (Emmelot *et al.*, 1959)) when isolated from " young " transplants, is less completely latent than in the case of rat liver mitochondria, the enzymic activity is, nevertheless, less pronounced and the effect of DNP more significant than with mitochondria isolated from " old " transplants from the same hepatoma. These differences are of interest in view of the results reported below.

* Recently Borst and Slater (1960) reported that heart mitochondria oxidize glutamate as α-ketoglutarate after an initial transamination with oxaloacetate, as shown *inter alia* by the complete (97 per cent) inhibition of glutamate oxidation in the presence of malonate. Glutamate oxidation by isolated mitochondria therefore does not constitute proof of the presence of glutamic dehydrogenase, since oxidation may proceed in the absence of the latter enzyme by the above mechanism. Compare the absence of the enzyme in the Novikoff hepatoma and the comment by Emmelot *et al.* (1959). In the present hepatoma mitochondrial preparations glutamate oxidation was inhibited for only 50 per cent by malonate, indicating the involvement of the glutamic dehydrogenase in the oxidation.

*Fatty acid oxidation*

The ability of the mitochondria from " young " transplants of the hepatoma BY 225, BY 446 and BY 463 to oxidize fatty acids (pH 7·4) is illustrated in Fig. 1 for hexanoate. For fatty acid oxidation to occur the fatty acids have first to be converted to their corresponding acylcoenzyme A derivatives (Lynen and Ochoa, 1953). This activation reaction needs ATP, and if the latter is missing or present below some critical level as a result of the poor phosphorylative efficiency (high ATPase activity) of the mitochondria, fatty acid oxidation will be impossible. Hence the failure to oxidize fatty acids need not be due to the absence of the enzymes of the $\beta$-oxidation cycle but may result from a lack of ATP.

It was, therefore, according to expectation to find that the mitochondria from the " old " transplants of the BY 225, BY 446 and BY 463 hepatomas, and those from " young " and " old " transplants of the BY 448 hepatoma, all showing low P : O ratios, were unable to oxidize fatty acids, in contrast to the mitochondria from " young " transplants of the former three hepatomas. Table IV contains a summary of these results. Since fatty acids may, to a variable extent depending on their chain length (Pressman and Lardy, 1956 ; Avi-Dor, 1960), induce mitochondrial ATPase activity, a disorganization that can be counteracted by serum albumin, hexanoate has been added in a number of experiments in the form of its albumin complex. To this end hexanoate was dissolved in a 5 per cent serum albumin solution which had been thoroughly dialyzed before use in order to remove oxidizable substrates. An aliquot thereof, corresponding to 2 $\mu$moles hexanoate and a final concentration of 0·3 per cent albumin was added to the respirometers. The presence of albumin, however, did not facilitate the hexanoate oxidation by

TABLE IV.—*Summary of Enzymic Properties of Mitochondria from Transplanted Rat Hepatomas*

In parentheses the experiments with " old " transplants.

| Hepatoma | Transplant generation | P : O ratio (glutamate oxidation) | Hexanoate oxidation |
|---|---|---|---|
| BY 252* | 45–60 | 2·2–2·5 | ++ |
| | [45–60 | .. | 0†] |
| BY 463 | 2 | 1·9 | ++ |
| | 3 | 2·5 | ++ |
| | [5 | 1·5 | 0] |
| BY 446 | [12 | 0 | 0] |
| | 13 | 1·6 | 0 |
| | 14 | 1·5 | 0 |
| | 15 | 2·4 | (albumin added) : + |
| BY 448 | [12 | 0·8 | 0] |
| | 13 | 0·4 | (albumin added) : 0 |
| | 14 | 0·7 | 0 |
| BY 484‡ | 2 | 1·9 | (albumin added) : ++ |
| | 4 | 2·6 | |

* Data from Emmelot *et al.* (1959).

† " Old " transplants of hepatoma BY 225, in contrast to " young " ones, were unable to oxidize octanoate ; hexanoate oxidation by mitochondria from the " old " transplants of this hepatoma has not been studied.

‡ Recent experiment not included in the text solid hepatoma (primary and transplanted).

the mitochondria from " young " BY 448 transplants, but did so with the BY 446 mitochondria which were otherwise not (very) active in oxidizing the fatty acid.

Since for the measurement of the P : O ratios and the fatty acid oxidation the same pools of mitochondria were used, the data summarized in Table IV suggest that for hexanoate activation (and oxidation) to be catalysed by a particular hepatoma-mitochondrial preparation the phosphorylation, accompanying the oxidation of glutamate in the presence of glucose-hexokinase, must at least exceed an efficiency corresponding to a P : O ratio of 1·6.

FIG. 1.—Hexanoate oxidation by mitochondria from the rat hepatomas BY 225, BY 463 and BY 446 (" young " transplants). Mitochondria from the same pools as used in the present experiments served also for the experiments illustrated in Table I. In one of the experiments (BY 446) serum albumin was present—see text. Incubation as described (Emmelot *et al.*, 1959) ; hexanoate (2 $\mu$moles), L-malate (0·0003 M), ATP (0·0007 M) and DPN (0·001 M) present in addition. Oxygen consumption corrected for that obtained in the absence of hexanoate. 0·82, 1·25 and 1·02 mg. mitochondrial nitrogen were present in the experiments with BY 225 (transplant generation : *60*), BY 463 (*2*) and BY 446 (*15*) mitochondria, respectively.

The data listed in Fig. 1 allow for a calculation of the maximum rates of hexanoate oxidation observed in the present systems by taking into account the oxygen uptakes during those time intervals in which oxygen consumption proceeded fastest (*e.g.* for BY 463 mitochondria between 10 and 20 minutes). The microatoms oxygen consumed/mg. mitochondrial N/hour, thus calculated, amounted to 33·3, 23·2 and 10·7 for the mitochondria from hepatoma BY 225, BY 463 and BY 446, respectively. For the complete oxidation of two $\mu$moles hexanoate to carbon dioxide and water (a trace amount of malate was always added) 347 $\mu$l. oxygen are required. This value is closely attained by the mitochondria from hepatomas BY 225 and BY 463 after a 3-hour period of incubation. The mitochondria from hepatoma BY 446 consumed only half of the theoretical amount of oxygen during this period, at the end of which the oxygen uptake had come to a stop. In the latter case, however, albumin was present and this condition, though protecting mitochondrial integrity, might hamper the availability of hexanoate to the mitochondria by withholding it in the albumin complex. The slower rate of hexanoate oxidation by the BY 446 mitochondria in the presence of albumin (Fig. 1) as compared to that by the other hepatoma mitochondria in the absence of albumin thus, at least partly, might be accounted for. Support for the

FIG. 2.—Palmitate oxidation by liver and hepatoma mitochondria. Incubation as indicated in Fig. 1, except that 0·4 μmole palmitate was now present. Dotted lines represent the oxygen uptake in the absence and solid lines in the presence of serum albumin (final concentration 0·3 per cent). Symbols :

● ········ ● = mouse liver mitochondria (1·08 mg. N),

• ——— • = mitochondria from rat hepatoma BY 252 (0·83 mg. N) and from mouse hepatoma T 28012 (1·04 mg. N),

▲ ——— ▲ = mitochondria from two primary rat hepatomas (0·77 mg. N).

latter explanation came from experiments with normal mouse liver mitochondria incubated with palmitate in the absence and presence of albumin (Fig. 2). Since, however, in the latter case with palmitate and albumin present, the oxygen consumption reached the non-albumin level after 100 minutes of incubation (not illustrated in Fig. 2), whereas in the case of the BY 446 mitochondria (Fig. 1) hexanoate oxidation in the presence of albumin stopped long before the theoretical amount of oxygen was consumed, it might be concluded that the latter mitochondria suffered damage during their incubation which resulted in a loss of fatty acid oxidation either by a fall in the ATP level or by the inactivation of the fatty acid oxidizing enzymes.

Palmitate oxidation by the hepatoma mitochondria has only been studied in the case of the hepatoma BY 225. In the absence of albumin no oxidation whatsoever was observed, but in the presence of albumin a distinct, though exceedingly small, oxygen uptake could be recorded ; a similar result was obtained with the mitochondria from the mouse hepatoma T 28012 (Fig. 2). As yet the only distinct evidence for palmitate oxidation by tumour mitochondria has been found in the case of two primary hepatomas induced by 4-dimethylaminoazobenzene in one rat (No. 391) of our inbred colony (Fig. 2). This result is mentioned here because it is, so far as we are aware, the first example to be presented for the ability of tumour mitochondria to oxidize palmitate.

*Activation of the $Mg^{2+}$-dependent and inhibition of the DNP-activated ATPase of rat liver mitochondria by non-structurally-bound lipids isolated from rat hepatomas*

In the introductory part of this paper it has been mentioned how we came to investigate the effect of the lipids, isolated from hepatoma homogenates, on the ATPase of normal rat liver mitochondria. After homogenisation of the tissues and application of the usual centrifugation for spinning down the nuclear fraction, the top-floating-lipid layer was collected from the tubes and washed several times by redispersion in fresh isotonic sucrose. Aliquots (0·1 ml.) of the final fatty dispersions were added to the incubation medium used for assaying the mitochondrial ATPase activity (Emmelot *et al.*, 1959). The concentration of the lipid was assessed by measuring the optical density at 520 m$\mu$ of 0·1 ml. of the final dispersion in 4·5 ml. sucrose. The latter values are listed in Table V together with the results of the ATPase assays carried out in the presence of $Mg^{2+}$ and with DNP ($10^{-4}$ M) absent or present. The effect of the lipids from the hepatomas BY 463, 446, 448 and normal rat liver on the ATPases of the normal rat liver mitochondria was tested at 27° C. and 37° C.

Lipid from rat liver (strain R-Amsterdam) showed little, if any, effect at 27° C. At 37° C. a slight increase in the $Mg^{2+}$-dependent and a small inhibition of the DNP-activated ATPase were observed (Table V). Since these effects were dependent on the presence of $Mg^{2+}$ it followed that they were mediated by an effect on the mitochondrial structure. Neither at 27° nor at 37° C. could any effect be observed from the addition of the lipid prepared from the hepatoma BY 463 (" young " transplant). However, when the lipid from an " old " transplant of the same hepatoma was used, a marked increase in the $Mg^{2+}$-dependent ATPase of the rat liver mitochondria was observed at a low concentration of the lipid. It is of interest that the hepatoma mitochondria obtained from this same transplant showed a high free ($Mg^{2+}$-dependent) ATPase activity, as illustrated in Table III, whereas the hepatoma mitochondria from non-necrotic " young " transplants showed a much lower activity. Given its small concentration, a distinct effect with the lipid from hepatoma BY 446 (young transplants) was obtained, it consisted of a small increase in the $Mg^{2+}$- and a small inhibition of the DNP-ATPase activity at 27° C and greater effects at 37° C. The lipid from hepatoma BY 448, added in the lowest concentration of all, activated the $Mg^{2+}$- and inhibited the DNP-ATPase to a marked extent ; the extra phosphate release normally brought about by DNP in the presence of $Mg^{2+}$ was completely abolished at 37° C. and almost so at 27° C.

The effect of various concentrations of the lipid from hepatoma BY 225 is illustrated in Table VI. It is seen that, whereas at 27° C. only the highest concen-

TABLE V.—*Effect of Liver and Hepatoma Lipids on the ATPase Activity of Rat Liver Mitochondria in the Absence and Presence of DNP*

Lipids obtained from " young " transplants, except for the case with BY 463 marked ¶. DNP: $10^{-4}$ M. Assay in the presence of $Mg^{2+}$, unless otherwise stated, as described previously (Emmelot *et al.*, 1959); 0·1 ml. of the lipid dispersion added to 1·8 ml. of the reaction mixture. Mitochondria from 25 mg. of fresh weight of rat liver (strain R) added except in the first experiment in which the equivalent from 40 mg. of liver was present.

| Source of lipid (concentration)* | Temperature of incubation (°C.) | μg. phosphor released: DNP absent after 7·5 min. | DNP absent after 15 min. | DNP present after 7·5 min. | DNP present after 15 min. |
|---|---|---|---|---|---|
| Control | 37 | 15 (4)† | 45 (21)† | 56 (83)† | 108 (130)† |
| Rat liver (0·090) | 37 | 29 (4)† | 58 (20)† | 50 (82)† | 75 (124)† |
| Control | 27 | 3 | 6 | 47 | 83 |
| BY 463 (0·155)‡ | 27 | 5 | 9 | 49 | 93 |
| Control | 27 | 3 | 7 | 50 | 80 |
| BY 446 (0·020) | 27 | 7 | 16 | 33 | 60 |
| BY 448 (< 0·020)‡‡ | 27 | 12 | 27 | 23 | 37 |
| Control | 37 | 10 | 15 | 94 | 120 |
| BY 463 (0·155)‡ | 37 | 10 | 15 | 87 | 120 |
| BY 463 (0·020)¶ | 37 | 25 | 48 | 66 | 105 |
| BY 448 (< 0·020)‡‡ | 37 | 44 | 62 | 48 | 65 |
| BY 446 (0·020) | 37 | 24 | 47 | — | — |

* Measured as indicated in the text.

† In the absence of $Mg^{2+}$.

‡ or ‡‡ Lipids from similar origin.

¶ Lipid from " old " transplant (generation: 5). The ATPase of the mitochondria from this transplant is illustrated in Table III.

TABLE VI.—*Effect of Various Concentrations of Lipid From Hepatoma BY 225 on the ATPase Activity of Rat Liver Mitochondria in the Absence and Presence of DNP*

0·1 ml. of the lipid dispersion in 4·5 ml. sucrose showed an optical density of 0·030 at 520 mμ.

| BY 225 lipid (ml. added) | Temperature of incubation (°C.) | μg. phosphor released: DNP absent after 7·5 min. | DNP absent after 15 min. | DNP present after 7·5 min. | DNP present after 15 min. |
|---|---|---|---|---|---|
| Control | 27 | 0 | 2 | 30 | 59 |
| 0·1 | 27 | 10 | 15 | 27 | 35 |
| 0·05 | 27 | 0 | 5 | 30 | 55 |
| 0·02 | 27 | 0 | 2 | 31 | 60 |
| Control | 37 | 4 | 7 | 62 | 124 |
| 0·1 | 37 | 26 | 53 | 39 | 65 |
| 0·05 | 37 | 18 | 50 | 60 | 100 |
| 0·02 | 37 | 7 | 22 | 60 | 110 |

tration of the lipid affected both the $Mg^{2+}$- and DNP-ATPase (activation and inhibition, respectively), all three concentrations of the lipid activated the $Mg^{2+}$-ATPase at 37° C., though with decreasing effect. At 37° C. the DNP-ATPase was markedly inhibited only by the lipid added in the highest concentration.

When the results obtained with the lipid from hepatoma BY 225 are compared with the data listed in Table V, it appears that the former lipid is about as active as that from hepatoma BY 446. It should be pointed out that in the present investigation no parallel experiments on the oxidative phosphorylation and fatty acid oxidation of the BY 225 mitochondria, such as has been the case with the other hepatomas, have been carried out. The results referred to above regarding the BY 225 hepatoma mitochondria have been obtained previously (Emmelot *et al.*, 1959), showing a certain degree of variation from one transplant to another.

TABLE VII.—*Histology and Some Enzymic Properties of Rat Hepatomas*

| Rat hepatoma | Primary tumour* | Transplants* | Mitochondrial oxidative phosphorylation and fatty acid oxidation† | dCMP‡ deaminase activity |
|---|---|---|---|---|
| BY 225 | Adeno—solid | Solid (fusiform cells) | " Normal " | High. |
| BY 446 | Adeno | Solid | Somewhat less than " normal " | Moderate—high. |
| BY 448 | Adeno—solid | Solid (few fusiform cells) | " Abnormal " | Moderate. |
| BY 463 | Solid | Solid | " Normal " | High. |
| ¶BY 484 | ,, | ,, | ,, | Moderate. |

* Emmelot *et al.* (1961).

† " Normal " = activities under optimal *in vitro* conditions, approximating those of rat liver mitochondria (differences : ATPase, release of diphosphopyridine nucleotide : Emmelot *et al.*, 1959).

‡ dCMP = deoxycytidylic acid, Emmelot *et al.* (1961).

¶ The dCMP deaminase activity of the 5th transplant generation of hepatoma BY 484 amounted to 37 and that of the 8th generation to 24–39 $\mu$moles of deoxyuridylic acid formed per equivalent of soluble fraction derived from 1 g. of fresh tissue/60 min. (units). Calculated per mg.N of soluble fraction, the activity ranged from 2·9 to 3·9 $\mu$moles deoxyuridylic acid formed per 60 min. Thymine-2-$^{14}$C catabolism was insignificant. Experimental conditions : Emmelot *et al.* (1961).

### DISCUSSION

*Attempt at classification of hepatomas.*—From the data summarised in Table VII it follows that no relation between the histological and enzymic properties of the transplants of the hepatomas can be established.

*Variability in hepatoma-mitochondrial behaviour.*—The following observations may constitute a basis for an explanation of the variability encountered in mitochondrial behaviour among the rat hepatomas.

(i) When the activities of the $Mg^{2+}$- and DNP-ATPases of normal liver mitochondria, hepatoma mitochondria and liver mitochondria incubated in the presence of hepatoma lipid, are compared, the non-latency of the $Mg^{2+}$- and the deficiency of the DNP-ATPase of the two latter types of particles, in contrast to the corresponding properties of the normal liver mitochondria, stand out (Emmelot *et al.*, 1959 ;

Emmelot and Bos, 1957—in the figures of the latter paper $P_i$ should be read as phosphor)

(ii) " Young " transplants of hepatoma BY 463 yielded mitochondria with low " free " ($Mg^{2+}$-) ATPase activity and a moderate DNP-activated ATPase, but " old ", necrotic, transplants yielded mitochondria with high " free " ATPase activity, DNP now causing hardly, if any, increment in ATP-splitting. The lipid obtained from the latter transplants activated the latent ATPase of normal liver mitochondria, in contrast to the lipid from the " young " transplants. Mitochondria from " old " transplants of BY 463 (and other) hepatomas, showed a poor oxidative phosphorylation and a lack of fatty acid oxidation.

(iii) The mitochondria from hepatoma BY 448 (" young " and " old " transplants) were deficient in fatty acid oxidation and in oxidative phosphorylation and exactly this hepatoma contained a lipid which was the most effective in activating the $Mg^{2+}$- and inhibiting the DNP-ATPase of normal liver mitochondria. The " young " transplants of hepatoma BY 463 contained a lipid devoid of any activity, whereas the mitochondria from this hepatoma showed a high oxidative phosphorylation and fatty acid oxidation. Finally, the BY 446 mitochondria were somewhat less efficient in the latter respects than the BY 463 mitochondria, and " young " transplants of the former hepatoma contained a lipid which was active in disturbing normal mitochondrial integrity, but less so than the lipid isolated from BY 448. Hence, a rather close parallel appeared to exist between the mitochondria-damaging effects of the lipids contained in the hepatomas and the activities of the mitochondria isolated from the corresponding hepatomas.

(iv) Complete removal of lipid droplets from the homogenates of hepatoma BY 225 has earlier been found (Emmelot *et al.*, 1959) to be required in order to obtain mitochondria with a relatively low " free " ATPase and a demonstrable DNP-ATPase.

(v) The hepatoma lipids were collected from the surface layer of the centrifuge tubes after centrifugation and thus represented non-structurally bound lipids (or lipoids, compare : Emmelot, Bos, and Reyers, 1960). By the very nature of this material,* the present results should be distinguished from those obtained by Pressman and Lardy (1956) and Avi-Dor (1960) with liver-microsomal fat or fatty acids and from those of Hülsmann, Elliott and Slater (1960) with unsaturated fatty acid released from liver mitochondria upon their ageing, though the mechanism of action might be the same in all cases. It might, however, be argued that our material, especially in the case of the " old " tumour transplants, contained breakdown products of mitochondrial and microsomal membranes.

In view of the above, we propose that adsorption of free lipids or related compounds from the hepatoma homogenates on to the mitochondria during the latter's isolation, may be responsible for the degree of lability inherent to the particles. This lability may range from very pronounced to slight, the present order being BY 448 $\gg$ BY 446 $>$ BY 463, as a result of the presence in the tumour cells of lipids possessing various degrees of mitochondria-damaging properties. The present hypothesis might also offer an explanation for the unsatisfactory behaviour of mitochondria isolated from " old " hepatoma transplants which contain much lipid material and possibly also for the variability encountered in the mitochondrial behaviour of other tumours (compare : Emmelot *et al.*, 1959).

* The present material designated as lipid has been found to contain fatty acids, cholesterol and some protein. The ATPase releasing activity is mainly residing in the long-chain fatty acid fraction.

Further support for the present conclusion may be adduced from previous experiments (Emmelot and Bos, 1957 ; Emmelot *et al.*, 1959—Table V, experiment marked*) which showed that in the presence of globulin the " free "—and DNP-ATPase of hepatoma and certain mouse liver mitochondrial preparations were decreased and enhanced, respectively, probably by binding of the lipid component. The sluggish or relatively poor effect of DNP on the hepatoma mitochondrial ATPase has led us earlier (Emmelot *et al.*, 1959) to postulate the possible existence of an endogenous inhibitor. The latter is now tentatively identified as to be of lipid nature, in view of the present results which demonstrated an inhibition of the DNP-activated ATPase by hepatoma lipid. The uncoupling agent found by Devlin and Pruss (1958) in the 25,000 $\times$ **g** supernatant of Novikoff hepatoma homogenates and apparently also in the mitochondria from this tumour, may also be of lipid nature.

Finally, we want to point out that in certain tumour homogenates the mitochondria may become contaminated by microsomal fragments—the phospholipide components (vesicles), containing high ATP- and DPN-splitting properties, which represent the double membranes of the endoplasmic reticulum *in situ*—especially in those cases in which high DPNase activities have been found inherent to certain tumour mitochondrial preparations (Emmelot, 1957 ; not in the case of BY 225 mitochondria : Emmelot *et al.*, 1959). Moreover, the possibility should be reckoned with that some tumour mitochondria *per se* possess a small intrinsic stability, a view that is supported by electron microscopical observations *in situ* (Emmelot *et al.*, 1959). Such particles might be especially vulnerable to the damaging effect of the lipids *in vitro* (if not already damaged by lipid *in situ*).

### SUMMARY

Mitochondria were isolated from " young " and " old " transplants of three rat hepatomas, BY 463, BY 446 and BY 448, and studied in respect of oxidative phosphorylation (substrate : glutamate) and fatty acid oxidation (substrate : hexanoate). The results were compared with those obtained previously with mitochondria from the rat hepatoma BY 225. With all four hepatomas the oxidative phosphorylation and the fatty acid oxidation was decreased or lacking when the mitochondria had been isolated from " old " transplants. Mitochondria from the " young " transplants, however, gave favourable results in the case of the hepatomas BY 225 and BY 463, and somewhat less so in the case of BY 446. By contrast, the mitochondria obtained from " young " transplants of the hepatoma BY 448 yielded P : O ratios always less than unity and were unable to oxidize hexanoate even if serum albumin was added. The latter two deficiencies are probably related in that fatty acid activation was made impossible by a lack of a sufficient amount of ATP. The BY 448 hepatoma contained a lipid which, in a low concentration, activated the $Mg^{2+}$-dependent and inhibited the dinitrophenol-activated ATPase of normal rat liver mitochondria to a marked extent. A smaller effect was displayed by the lipid from hepatoma BY 446 but none at all by that from hepatoma BY 463. However, lipid isolated from " old " transplants of the latter tumour activated the latent ATPase of liver mitochondria and hepatoma mitochondria isolated from the latter transplants possessed high " free " ATPase activities, in contrast to the low to moderate free ATPase activities of BY 463 hepatoma mitochondria from " young " transplants. An adsorption of the active lipids from the particular hepatoma homogenates onto the mitochondria during the latter's isolation, followed by structural and functional disarrangement, might account for the lability noted among the corresponding mitochondrial preparations. The latter problem is briefly discussed.

### REFERENCES

Avi-Dor, Y.—(1960) *Biochim. biophys. Acta*, **39**, 53.
Borst, P. and Slater, E. C.—(1960) *Ibid.*, **41**, 170.
Devlin, T. M. and Pruss, M. P.—(1958) *Fed. Proc.*, **17**, 211.
Emmelot, P.—(1957) *Exp. Cell. Res.*, **13**, 601.
*Idem* and Benedetti, E. L.,—(1960) *J. biophys. biochem. Cyt.*, **7**, 393.
*Idem* and Bos, C. J.—(1957) *Brit. J. Cancer*, **11**, 148.
*Idem*, Bos, C. J., Brombacher, P. J. and Hampe, J. F.—(1959) *Ibid.*, **13**, 348.
*Idem*, Bos, C. J., Brombacher, P. J. and Reyers, I. H. M.—(1960) *Nature, Lond.*, **186**, 556.
*Idem*, Bos, C. J. and Reyers, I. H. M.—(1960) *Z. Krebsforsch.*, **64**, 52.
*Idem*, Hampe, J. F., Bos, C. J. and Reyers, I. H. M.—(1961) *Brit. J. Cancer*, **15**, 138.
Hülsmann, W. C., Elliott, W. B. and Slater, E. C.—(1960) *Biochem. biophys. Acta*, **39**, 267.
Lynen, F. and Ochoa, S.—(1953) *Ibid.*, **12**, 299.
Pitot, H. C. and Potter, V. R.—(1960) *Ibid.* **40**, 537.
Pressman, B. C. and Lardy, H.—(1956) *Biochim. biophys. Acta.* **21**, 458.


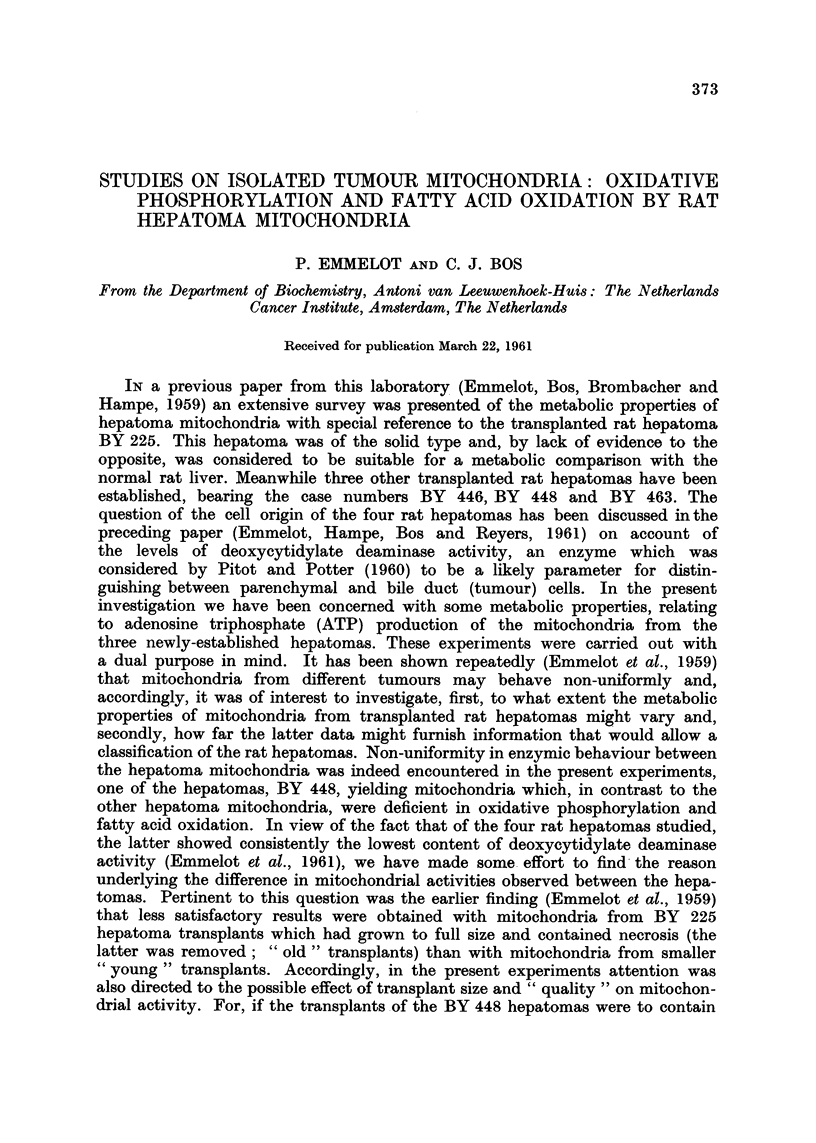

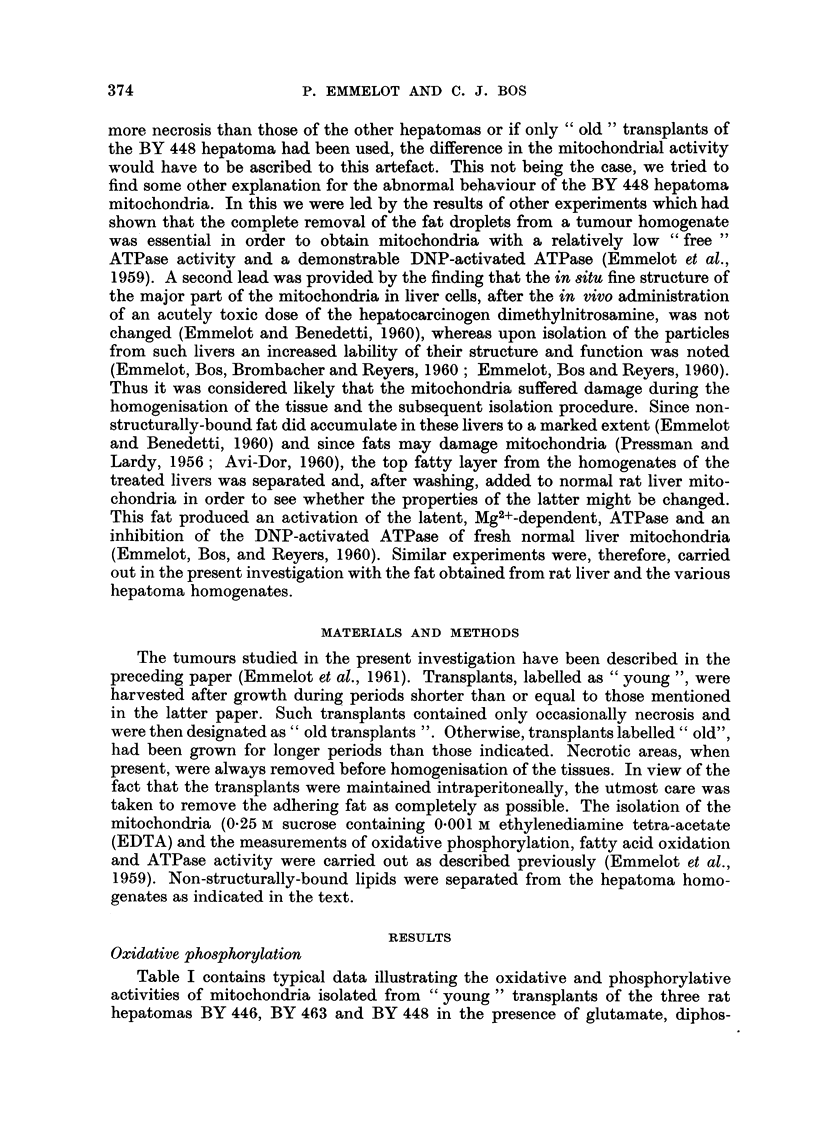

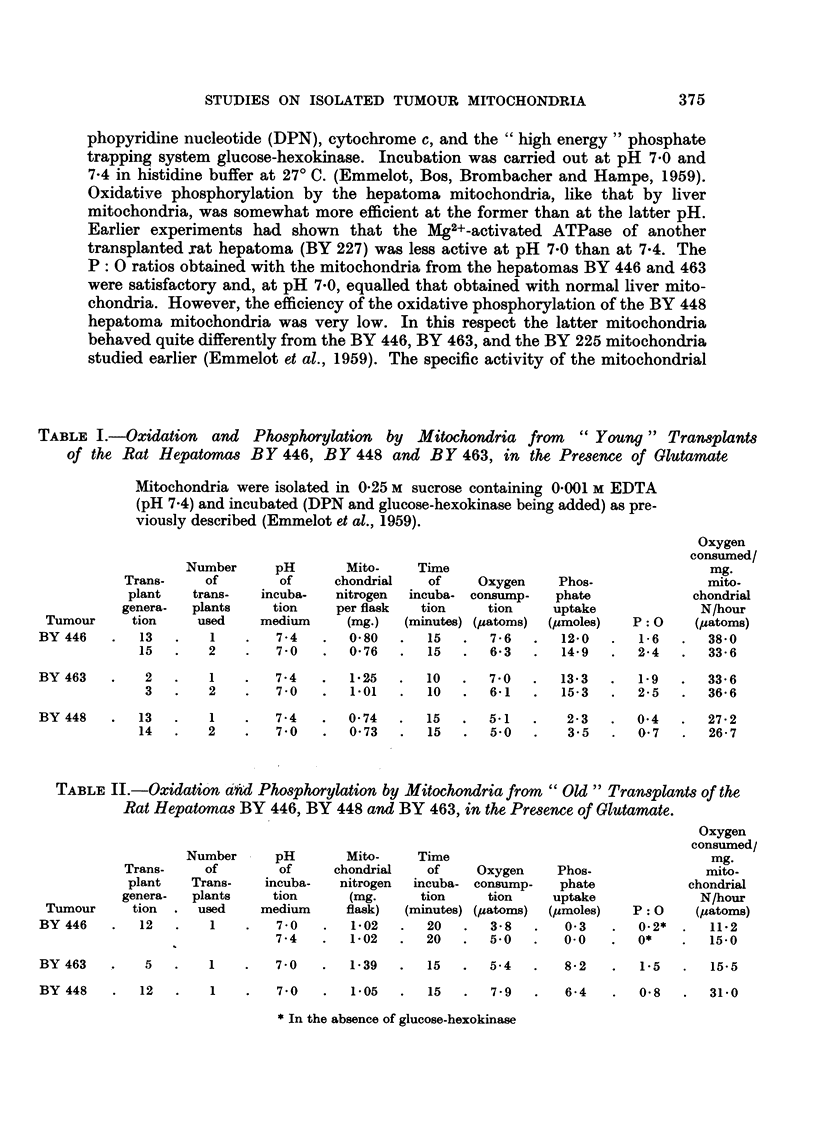

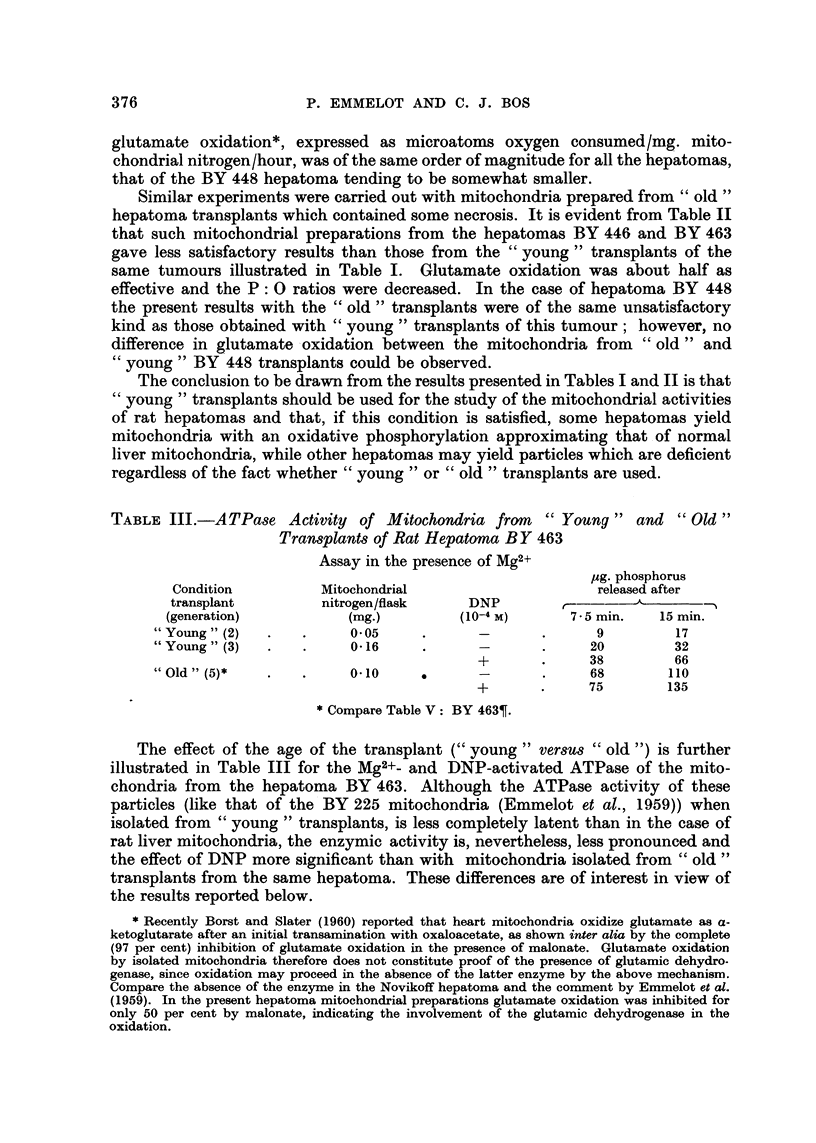

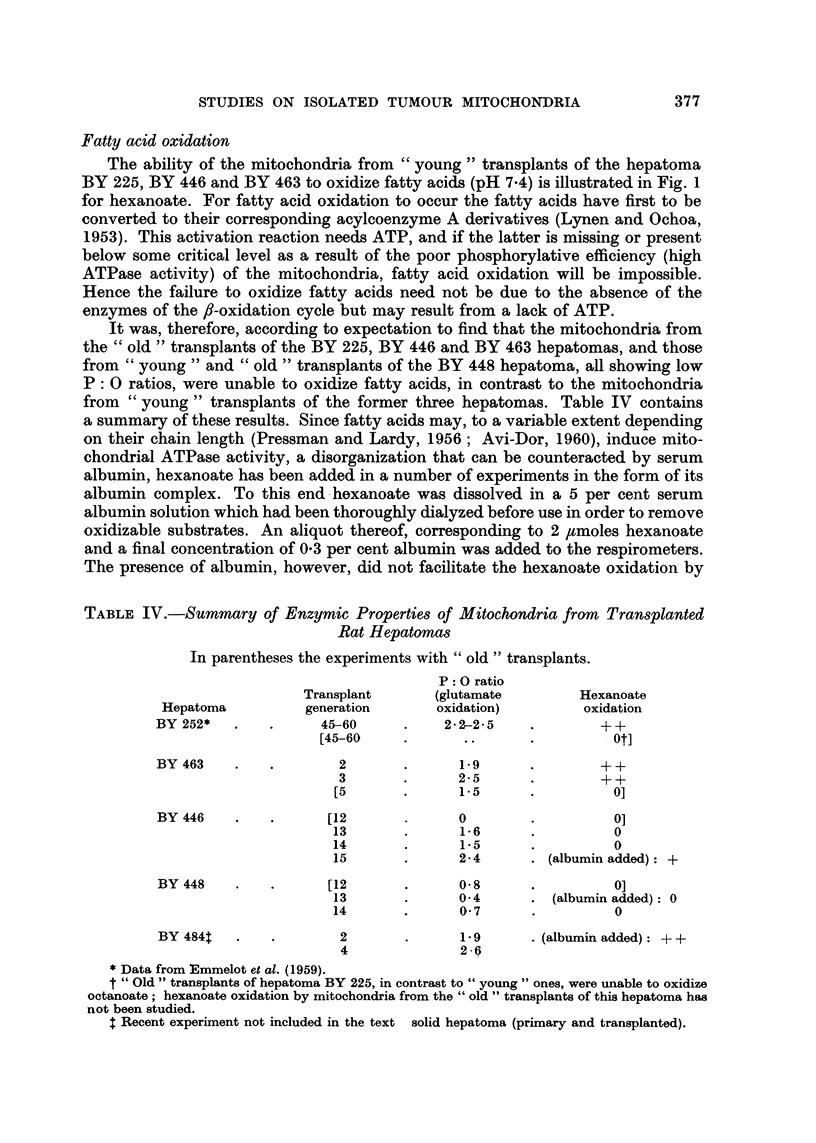

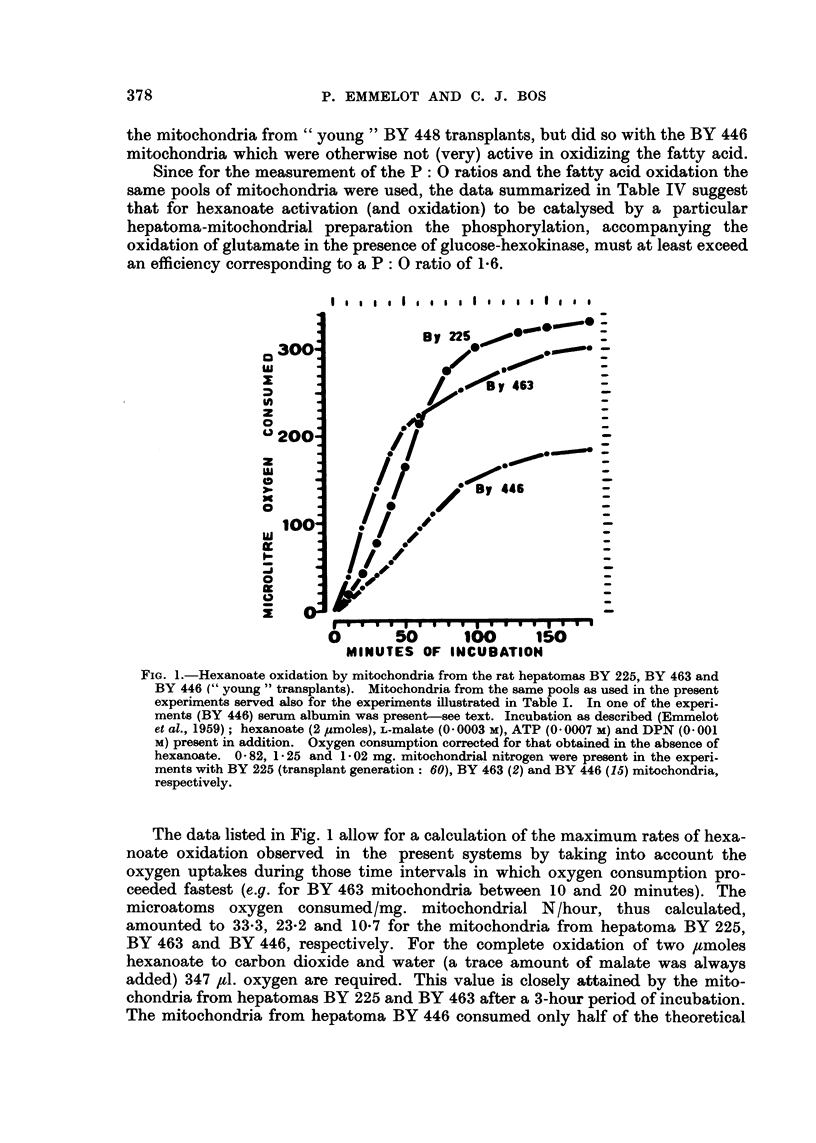

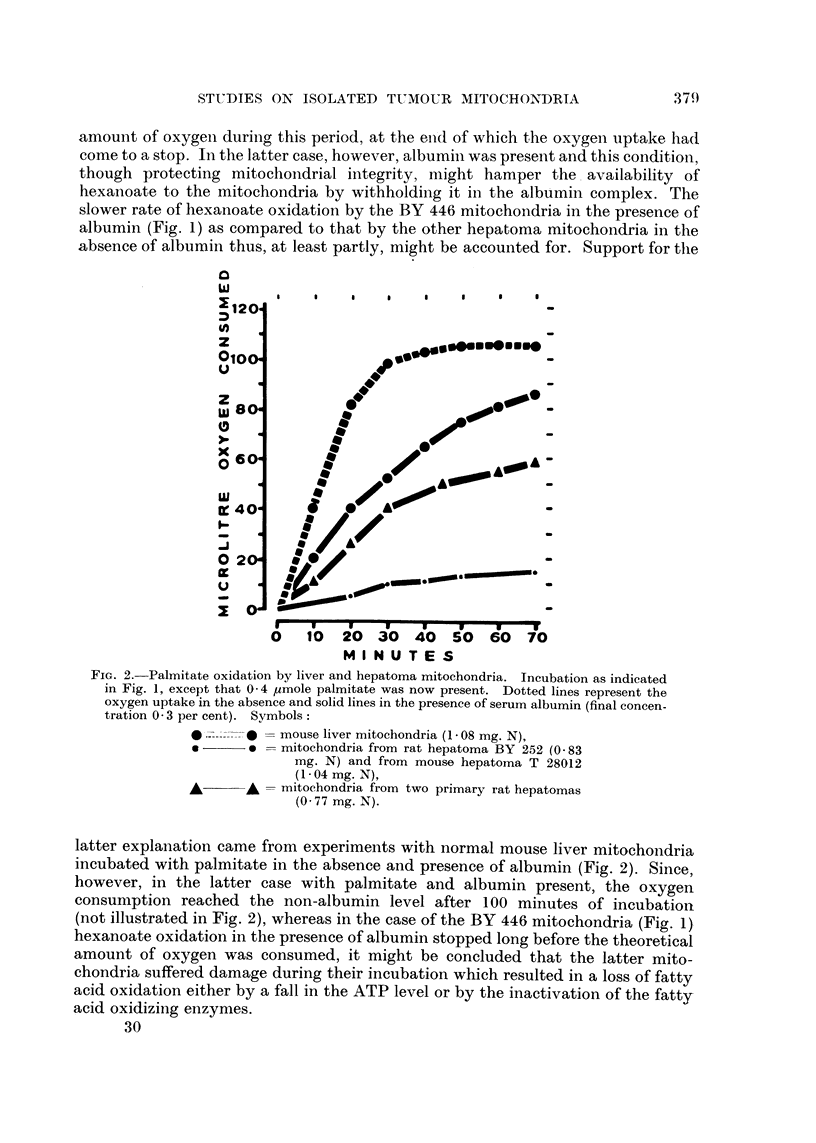

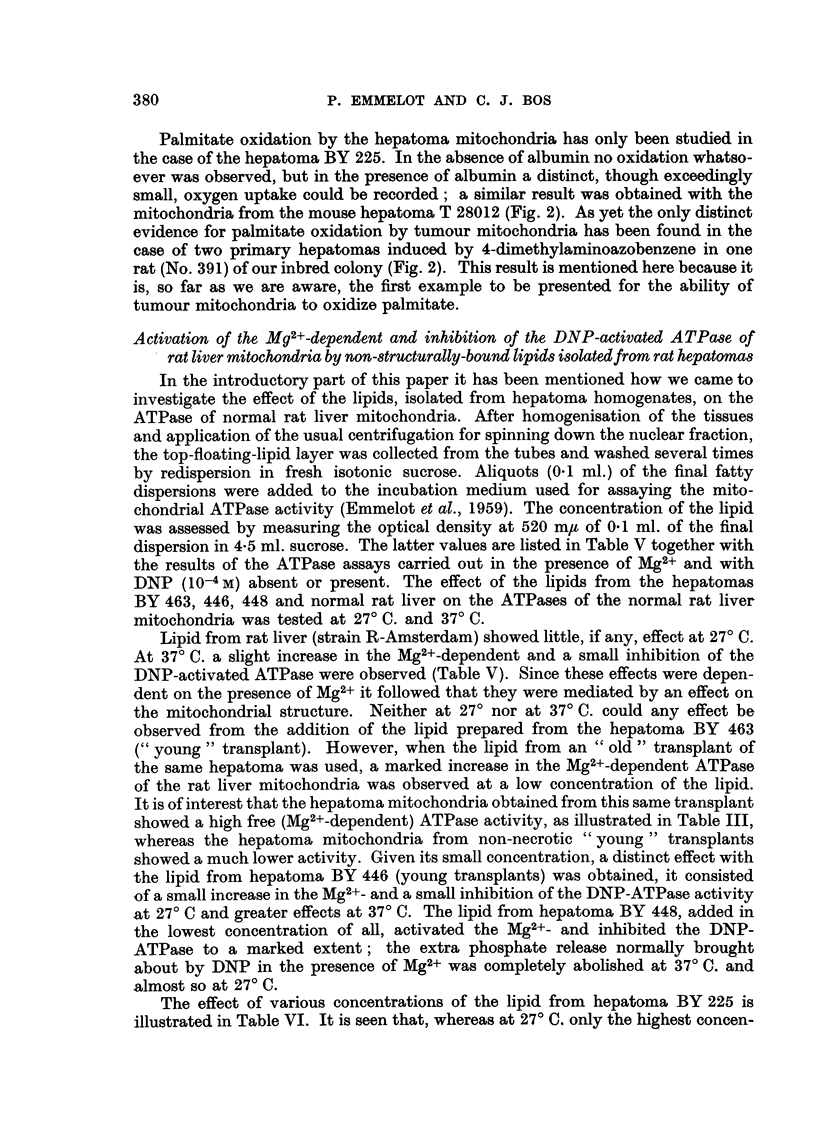

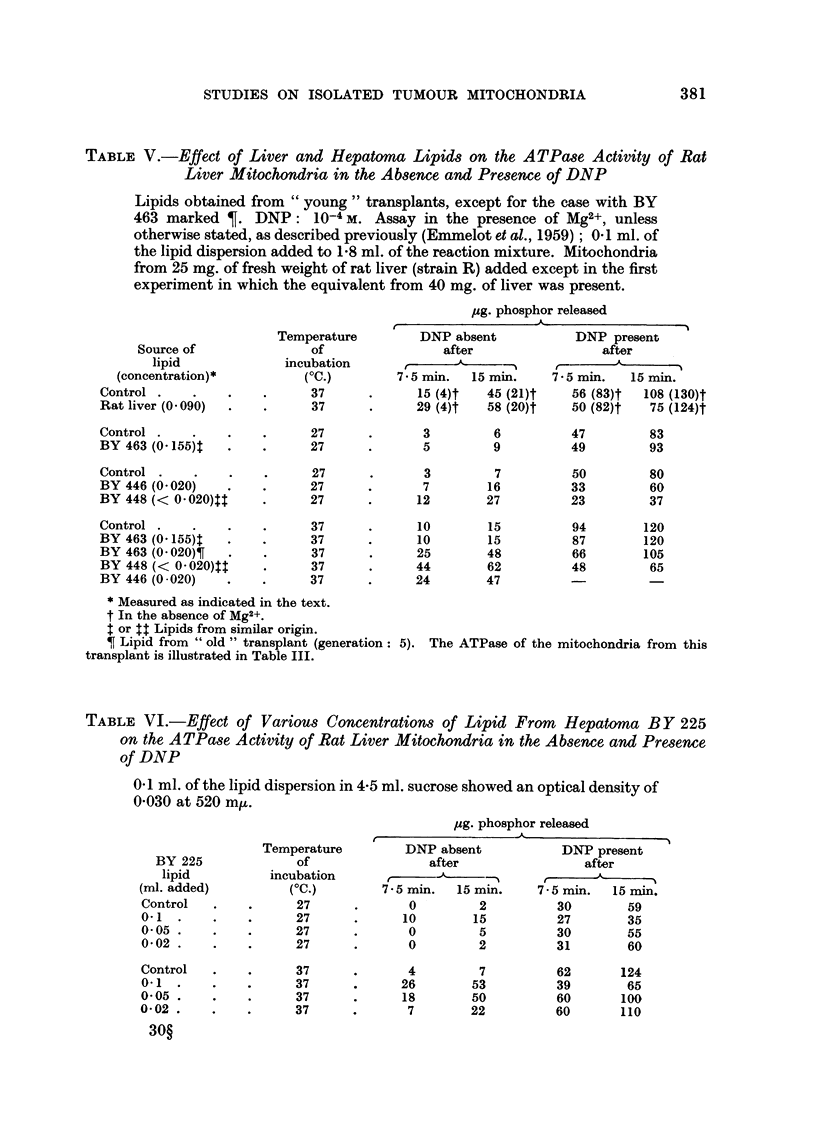

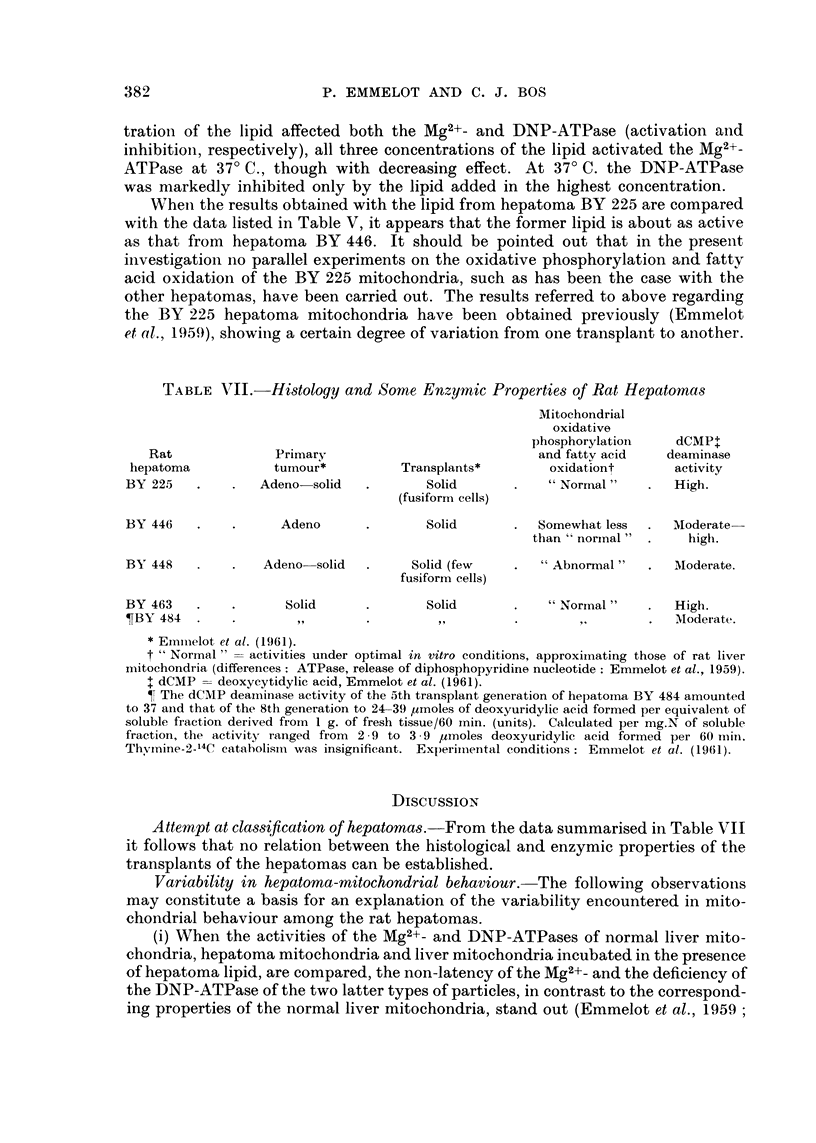

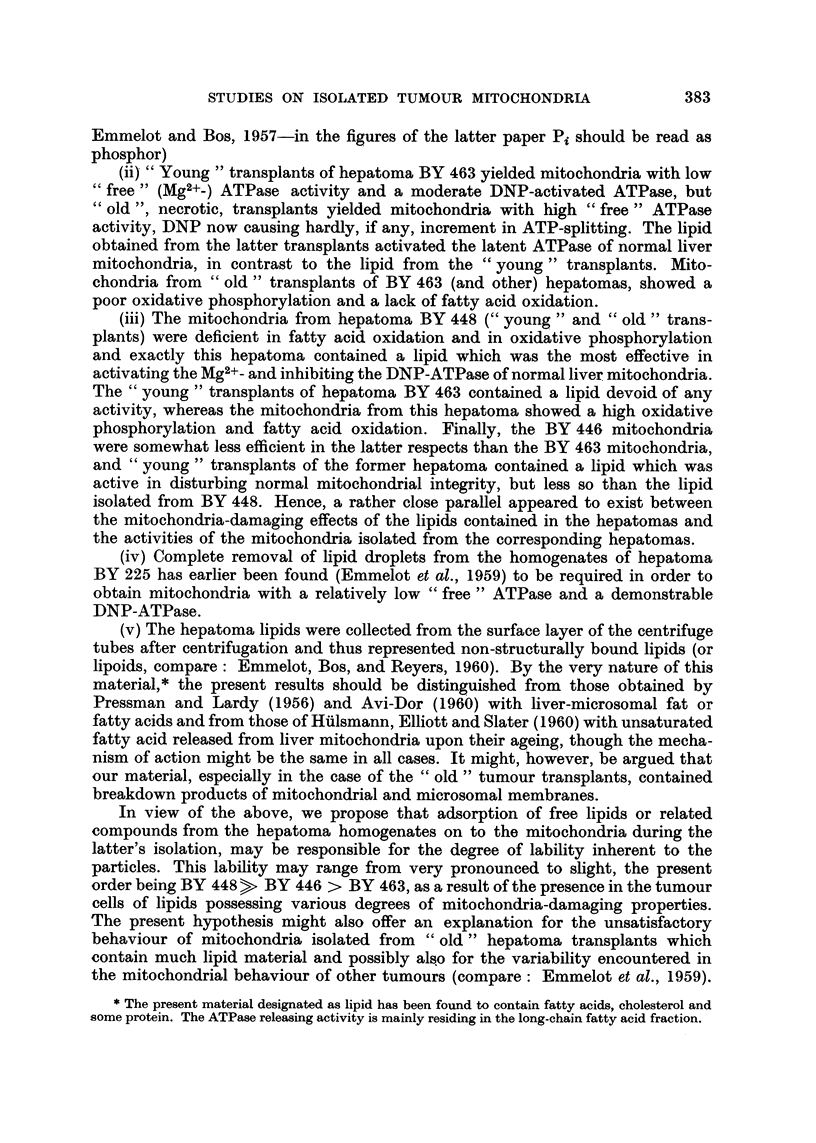

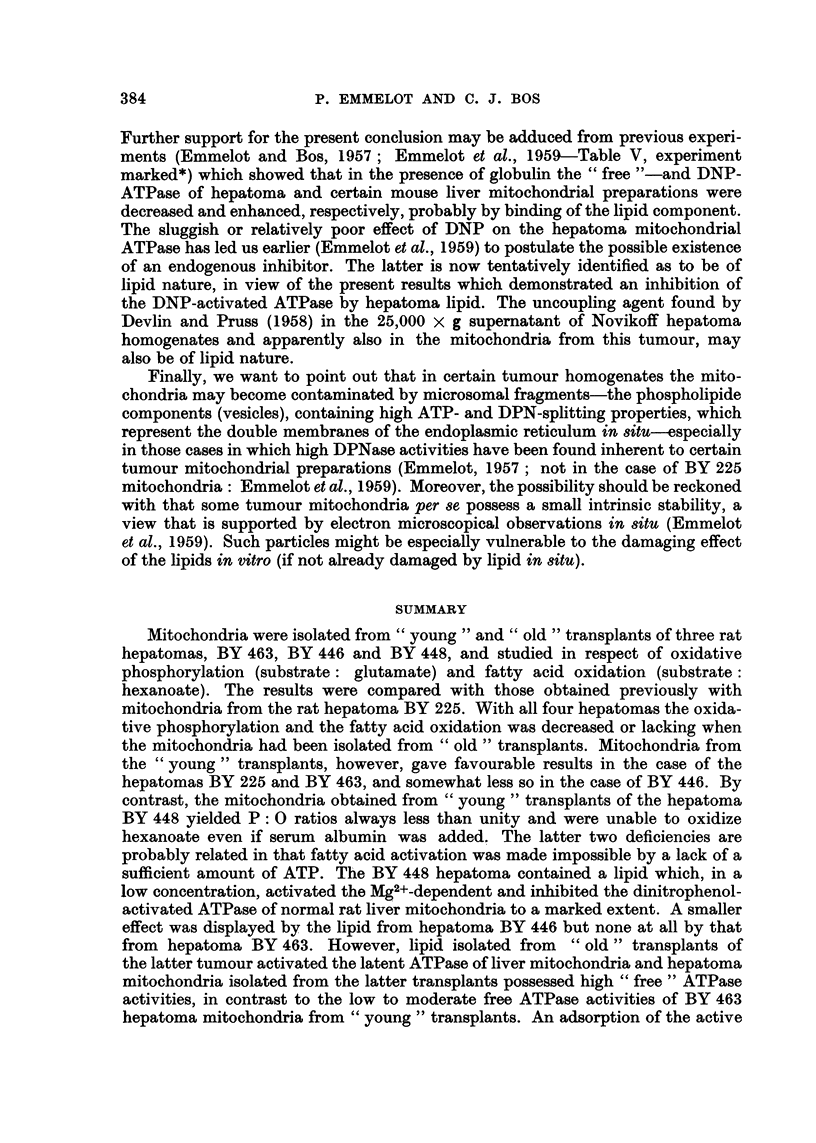

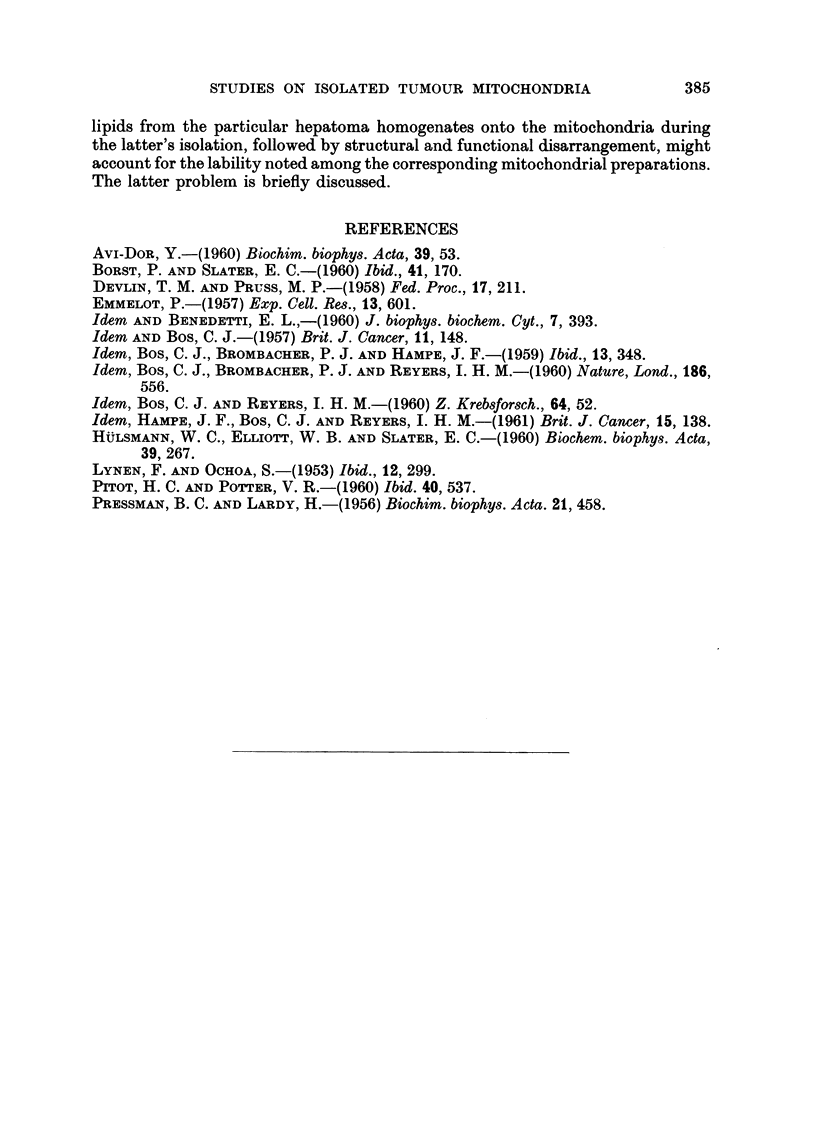


## References

[OCR_00938] BORST P., SLATER E. C. (1960). The oxidation of glutamate by rat-heart sarcosomes.. Biochim Biophys Acta.

[OCR_00519] EMMELOT P., BOS C. J. (1959). The metabolism of neoplastic tissues: the effect of 2:4-dinitrophenol on the respiration of ascites tumour cells.. Br J Cancer.

[OCR_00941] EMMELOT P. (1957). The in vitro incorporation of C14-leucine into the proteins of tumor microsomes and a note on the properties of isolated tumor mitochondria.. Exp Cell Res.

[OCR_00963] LARDY H. A., PRESSMAN B. C. (1956). Effect of surface active agents on the latent ATPase of mitochondria.. Biochim Biophys Acta.

[OCR_00959] LYNEN F., OCHOA S. (1953). Enzymes of fatty acid metabolism.. Biochim Biophys Acta.

[OCR_00961] PITOT H. C., POTTER V. R. (1960). An enzymic study on the cellular origin of the Dunning and the Novikoff hepatomas in the rat.. Biochim Biophys Acta.

